# Editorial: Epigenetic and Related Signaling Pathways in Response to Ionizing Radiation and Nano-Particles

**DOI:** 10.3389/fcell.2022.932757

**Published:** 2022-05-27

**Authors:** Ruixue Huang, Qunwei Zhang, Pingkun Zhou

**Affiliations:** ^1^ Xiangya School of Public Health, Central South University, Changsha, China; ^2^ Department of Environmental and Occupational Health Sciences, School of Public Health and Information Sciences, University of Louisville, Louisville, KY, United States; ^3^ Department of Radiation Biology, Beijing Key Laboratory for Radiobiology, Beijing Institute of Radiation Medicine, AMMS, Beijing, China

**Keywords:** signaling pathways, ionizing radiation, nano-particles, co-effect, epigenetic

With the development of our society and technology, ionizing radiation (IR) and nano-particles (NP) have been widely included in the medical therapies. In respect of the toxicity of IR and NP, they are considered as potential health risks (Huang, 2021). Increasing evidence from experiments using animal models and cell reported that IR and NP can induce extensive DNA damage or genomic instability that has serious consequences for cells and organisms leading to the development of pathologies like cancer (Huang and Zhou, 2021). Furthermore, NP has been reported to be potential toxicological effects on cells thus represent a major concern for human population health (Lei et al., 2022). In-depth studies on the molecular mechanisms have indicated that epigenetic and related signaling pathways may play critical roles in the response to IR and NP. For example, more and more studies have mushroomed to demonstrate that microRNAs, long-coding RNAs (lncRNAs) and circle RNAs (circRNAs) can regulate or be regulated by IR and NP (Zhang et al., 2020). Many signaling pathways such as DNA damage repair pathways can be affected, and many repair-related genes and proteins may be involved in the regulation of IR and NP responses (Huang and Zhou, 2020). The outcome of these responses primarily depends upon the level and type of IR and NP as well as the genetic background of the exposed cell or organism and epigenetic factors (Huang and Zhou, 2019).

The present Research Topic hosts an overview by Ruixue Huang, Qunwei Zhang, and Pingkun Zhou on the pathways in response to ionizing radiation and nano-particles. As discussed by the authors, IR and NPs often threaten human health in combinational status. Moreover, as IR and NP are commonly exposed in the environment, industrial activities and medical applications, their combinational effects on human health should be considered.

The observations derived from Liang et al. indicate that inhibition of PRMT5 has also become a potential therapy for methionine adenosine phosphorylase (MTAP)-deficient cancers. Liu et al. reveal that ESR1 promoted SLC7A11 expression at the early stage after IR. ESR1/SLC7A11 knockdown significantly enhanced IR-induced ferroptosis in ER-positive cells. Jia et al. reviewed the roles of microRNAs in the IR and point out the critical potential of microRNA in the regulation of IR response. Xu et al. demonstrated that the miR-122-5p containing-EVs derived from hypoxic HIEC cells promoted apoptosis in normoxic HIEC cells. Hypoxic EV-derived miR-122-5p plays a critical pathologic role in radiation-induced rectal injury and may be a potential therapeutic target. He et al. reported that environmental folic acid may modulate lipid metabolism through activating AMPK signaling pathway.

Overall, the article collection in this Research Topic provides a comprehensive overview of several regulatory signaling pathways and targets in response to IR or NPs, describing well-consolidated effects, and discussing more recent findings highlighting the key regulator role of microRNA in IR-related disease, such as cancer.
